# Dysregulated miRNAs as Biomarkers and Therapeutical Targets in Neurodegenerative Diseases

**DOI:** 10.3390/jpm12050770

**Published:** 2022-05-10

**Authors:** Giulia Gentile, Giovanna Morello, Valentina La Cognata, Maria Guarnaccia, Francesca Luisa Conforti, Sebastiano Cavallaro

**Affiliations:** 1 Institute for Biomedical Research and Innovation, Department of Biomedical Sciences, National Research Council (CNR), Via Paolo Gaifami, 18, 95126 Catania, Italy; giulia.gentile@cnr.it (G.G.); giovanna.morello@irib.cnr.it (G.M.); valentina.lacognata@irib.cnr.it (V.L.C.); maria.guarnaccia@cnr.it (M.G.); 2 Medical Genetics Laboratory, Department of Pharmacy, Health and Nutritional Sciences, University of Calabria, Via Pietro Bucci, Arcavacata, 87036 Rende, Italy; francescaluisa.conforti@unical.it

**Keywords:** *post-mortem* human tissues, iPSC-derived neurons, circulating fluids, AD, PD, ALS, ASOs-based therapies, drug biomarkers, miR-124, miR-218

## Abstract

Alzheimer’s disease (AD), Parkinson’s disease (PD), and Amyotrophic Lateral Sclerosis (ALS) are representative neurodegenerative diseases (NDs) characterized by degeneration of selective neurons, as well as the lack of effective biomarkers and therapeutic treatments. In the last decade, microRNAs (miRNAs) have gained considerable interest in diagnostics and therapy of NDs, owing to their aberrant expression and their ability to target multiple molecules and pathways. Here, we provide an overview of dysregulated miRNAs in fluids (blood or cerebrospinal fluid) and nervous tissue of AD, PD, and ALS patients. By emphasizing those that are commonly dysregulated in these NDs, we highlight their potential role as biomarkers or therapeutical targets and describe the use of antisense oligonucleotides as miRNA therapies.

## 1. Introduction

Neurodegenerative diseases (NDs) selectively affect distinct brain regions and neuronal types with different molecular processes and the aggregation of misfolded proteins [1]. This is the case of Alzheimer’s disease (AD) [2], Parkinson’s disease (PD) [3], and Amyotrophic Lateral Sclerosis (ALS) [4].

AD represents the most common ND of aging and the leading cause of dementia worldwide and is characterized by the accumulation of amyloid-β (Aβ) and tau aggregates in different brain areas [2,5]. PD is the most common neurodegenerative movement disorder and is characterized by the loss of dopaminergic neurons (DNs) in *substantia nigra pars compacta* (SNpc) and the accumulation of toxic amyloid structures made up of α-synuclein aggregates [3,6]. ALS, also known as Lou Gehrig’s disease, represents a progressive neurodegenerative disease of adulthood and is due to the progressive degeneration of upper and/or lower motor neurons (MNs) and, in some cases, by ubiquitinated protein aggregates [4,7]. Even if some treatments are able to alleviate symptoms or prolong life expectancy, there is still no cure for these NDs [8,9,10,11,12,13] and the primary goal today is the identification of effective therapies. The development of new treatment options requires a better understanding of the molecular basis underlying these pathological conditions and the identification of sensitive and specific disease biomarkers to aid early diagnosis and monitor disease progression and response to treatment.

Different non-coding RNAs have been proposed as biomarkers of neurodegeneration and, among them, microRNAs (miRNAs) have attracted the scientific community’s attention thanks to their role as key regulators of gene expression [14,15,16,17]. MiRNAs are short molecules (20–22 nucleotides) able to degrade or inhibit the translation of their multiple complementary mRNA targets in a cell- and tissue-specific manner [17]. Common target sites for endogenous miRNAs are located in the 3′UTR region of mRNAs where they form an imperfect duplex hybrid and regulate their translation [18]. Their role in the nervous system is not limited to cells where they are produced, but numerous extracellular miRNAs are released and exchanged in a cross-talk between blood, cerebrospinal fluids (CSF), brain, and periphery [19].

Several miRNAs were found dysregulated in human pathology and animal models of NDs, supporting their role as disease biomarkers. More recently, the molecular and functional overlapping of dysregulated miRNAs has been reported in different NDs [15]. Due to their increasing importance in pathology, miRNA-based therapeutic strategies are also gaining interest. Indeed, miRNA suppression or replacement by antisense oligonucleotides (ASOs) technologies can be successfully used in animal models or in patients with NDs [9,10,11,13,20].

While fluids, such as plasma, serum, or CSF offer the possibility to monitor drug effects by the expression of biomarkers during the onset/progression of NDs, changes observed in nervous tissues are fundamental to define their direct or indirect implication in neurodegeneration [21]. The analysis of miRNAs in both fluids and nervous tissues may help to characterize their dynamic inter-communication between periphery (blood and organs) and brain (blood and brain, blood and CSF, CSF and brain) [19] and prioritize their selection as disease biomarkers and therapeutical targets.

To better investigate the role of miRNAs or their targets in the pathogenesis of NDs and evaluate their potential application as biomarkers, here we review miRNAs that were found dysregulated (in at least two independent studies) in *post-mortem* nervous tissue, as well as fluids of patients affected by AD, PD, and ALS. By emphasizing those that are commonly dysregulated in these NDs, we highlight their potential role as biomarkers or therapeutical targets and describe the use of antisense oligonucleotides as miRNA therapies.

## 2. AD

AD represents the most common age-related neurodegenerative disorder and is characterized by the presence of β-amyloid-containing plaques and tau-containing neurofibrillary tangles (NFTs) in different brain districts. The majority of cases manifest as a late-onset sporadic form (sAD), whereas familial forms (fAD) are mainly due to pathogenic variants in *APP*, *PSEN1,* and *PSEN2* [22]. From a molecular perspective, AD is characterized by extracellular deposits of Aβ peptides, generated in the amyloidogenic pathway from the cleavage of APP by BACE1 and γ-secretase, and by the intracellular accumulation of strings of hyperphosphorylated Tau proteins known as neurofibrillary tangles (NFTs) [23]. In particular, Aβ peptides accumulation is due to the unbalanced synthesis and clearance of Aβ oligomers, and the mechanisms involved in Aβ clearance include ubiquitin–proteasome system (UPS), autophagic processes, proteolytic regulation and clearance of blood-brain barrier (BBB) [24].

As shown in Table 1, 17 miRNAs (miR-7, miR-9, miR-16, miR-29a, miR-29b, miR-32, miR-34a, miR-34c, miR-101, miR-124, miR-125b, miR-128, miR-132, miR-135a, miR-146a, miR-195, and miR-218) were found dysregulated by at least two independent studies in different brain regions and fluids of AD patients. Four of them (miR-9, miR-124, miR-125b, and miR-195) were also implicated in AD iPSC-derived neurons (Table 1).

**Table 1:** Dysregulated miRNAs in human AD *post-mortem* tissues and circulating fluids.

miRNAs	AD *post-mortem* CNS/AD iPSC-Derived Neurons	Validated Target	Signaling Pathway	Circulating Fluids
miR-7	Up-regulated in hippocampus [25,26], entorhinal cortex, middle temporal gyrus, posterior cingulate cortex, superior frontal gyrus [26], and cortex [27]; down-regulated in grey matter [28], anterior cingulate gyrus (Brodmann area 24), motor cortex [29], and temporal cortex [30]	*UCHL1* [31]; *UBE2A* [32]	Ubiquitin-mediated clearance of amyloid peptides mediated by ciRS-7 [32]; NF-κB-dependent regulation of APP and BACE1 protein and degradation by proteasome and lysosome through UCHL1 [31]; insulin signaling through HNRNPK–miR-7 axis [27]	Detected in peripheral blood [33]
miR-9	Down-regulated in the anterior temporal cortex [34], grey matter [28], cerebellum, hippocampus, medial frontal gyrus [25], and temporal cortex [30]; up-regulated in hippocampal CA1 region [35], and temporal lobe neocortex (Brodmann area A22) [36]; used to obtain a rapid neuronal differentiation and an AD disease phenotypes detected at early time points due to rapid maturation of iPSCs [37]	*BACE1* [34]; *CREB* [38]; *OPTN* [39]; *CAMKK2* [40]; *TGFBI*, *TRIM2*, *SIRT1* [41]	miR-9 mediates the expression of BACE1 by directly regulating CREB [38]; autophagy [39]; CAMKK2-AMPK2 pathway [40]	Down-regulated in whole blood of LOAD patients [42]; CSF decreasing with increasing of Braak stages [43]; up-regulated in exosome enriched CSF [44]
miR-16	Down-regulated in white matter [28], and Braak VI hippocampus [45]; up-regulated in Braak III/IV	*APP* [46]; *TAU1* [47]		Decreasing with the increasing of Braak stages in serum [43]; down-regulated in CSF [48]
miR-29a	Down-regulated in the anterior temporal cortex [34], medial frontal gyrus [25];, and grey matter [28]	*BACE1* [34]	BACE1/β-secretase expression [34]	Up-regulated in CSF [49], and cell-free CSF [50]; down-regulated in CSF [48]
miR-29b	Down-regulated in anterior temporal cortex [34], parietal lobe cortex [51], grey matter [28], dorsolateral prefrontal cortex (Brodmann area 9) and temporal cortex (Brodmann area 21/22) [52]; up-regulated in medial frontal gyrus [25]	*BACE1* [34]	BACE1/β-secretase expression [34]	Up-regulated in CSF [49]
miR-32	Down-regulated in the cerebellum, hippocampus, medial frontal gyrus [25], and white matter [28]	*MECP2* [53]	Feedback loop with MeCP2 and BDNF for homeostatic regulation of MeCP2 [53]	Up-regulated in CSF [25], and in serum [30]
miR-34a	Up-regulated in cerebellum, hippocampus, medial frontal gyrus [25], hippocampal CA1 [54], anterior cingulate gyrus (Brodmann area 24) and motor cortex [29]; down-regulated in grey matter [28]	*TREM2* [54]; *SHANK3* [55]	Synaptogenesis and phagocytosis [54,55]	Down-regulated in plasma and CSF [49]
miR-34c	Down-regulated in white matter [28]; up-regulated in the hippocampus [56], Braak stage III/IV hippocampus [45], anterior cingulate gyrus (Brodmann area 24), and motor cortex [29]	*SIRT1* [56]		Up-regulated in serum [43]
miR-101	Down-regulated in white matter [28], anterior temporal cortex [34], and parietal lobe cortex [51]	*APP* [57]	IL-1β-induced APP up-regulation [57]	Down-regulated in CSF [43]
miR-124	Down-regulated in gray matter [28], frontal cortex [58], temporal cortex [30]; up-regulated in iPSC-derived iNEU-PSEN hippocampal neuron from the AD patient [59]	*BACE1* [58,60]; *PTPN1* [61,62]; *APP* [59]	*PTPN1* signaling [61]	Down-regulated in CSF [43]
miR-125b	Up-regulated in hippocampal CA1 region [35,54], temporal lobe neocortex (Brodmann area A22) [36], cerebellum, hippocampus, medial frontal gyrus [25], frontal cortex (Brodmann areas 6 and 8) [63], iPSC-derived iNEU-PSEN hippocampal neuron from the AD patient [59], and APP and PS1 variants of hippocampal spheroids differentiated from iPSC (3D hippocampal structures) [64]; down-regulated in grey matter [28]	*CFH* [65]; *DUSP6, PPP1CA; BCLW* [63]; *CDKN2A* [66]; *NR2A* [67]	*CFH*-driven pathogenic signaling [65]; miR-125b-induced tau hyperphosphorylation [63]; astrogliosis and glial cell proliferation [66]; FMRP-associated up-regulated miRNA induces long narrow spines [67]	Down-regulated in CSF [48,49]; up-regulated in CSF [68]
miR-128	Up-regulated in hippocampal CA1 [35,55], Braak III/IV and decreased in Braak VI hippocampus [45], and temporal cortex [30]; down-regulated in cerebral cortical gray matter [28], and hippocampus of LOAD patients [69]	*PPARG* via regulation of the NF-κB pathway [70]	NF-κB pathway [70]	Up-regulated in monocytes and lymphocytes from AD patients [71]
miR-132	Up-regulated in hippocampal CA1 region [35,55], anterior cingulate gyrus (Brodmann area24) and motor cortex [29]; down-regulated in cerebellum, medial frontal gyrus [25], temporal cortex [30,72], frontal cortex [72], prefrontal cortex [73], olfactory bulb [74], hippocampus [25,72,73,74], and hippocampus and prefrontal cortex of LOAD [69]	*P250GAP* [75]; *PTBP2* [76]; *HDAC3* [77]; tau levels [72]; *ITPKB* [73]; *SIRT1* [74]; *HNRNPU* [78]	FMRP-associated up-regulated miRNA increases dendritic protrusion width [67]; miR-132/ITPKB pathway [73]; CREB-regulated miRNA regulates neuronal morphogenesis [75]; HDAC3 signaling pathway [77]; hippocampal pro-neurogenic signal rescue [79]	Down-regulated in CSF [43]; up-regulated in plasma [80]
miR-135a	Up-regulated in hippocampus [25], anterior cingulate gyrus and motor cortex [29]; down-regulated in gray matter [28], and frontal cortex [81]	*BACE1* [82]; *THBS1* [83]	CEBPD/miR135a/THBS1 axis promotes angiogenesis [83]; Rock2/Add1 signaling pathway-miRNA regulated mediates the synaptic/memory impairments [81]	Up-regulated in CSF [25], serum [43], and exosomal serum [84]
miR-146a	Up-regulated in hippocampal [85,86] and superior temporal lobe neocortex [36,85,86], hippocampal CA1 [54,55], Braak III/IV and decreased in Braak VI hippocampus [45]; down-regulated in temporal cortex [30]	*CFH* [65,85]; *IRAK-1* and *IRAK-2* [86,87]; *SHANK3* [55]; Srsf6 [88]	Altered innate immune response and neuroinflammation through *CFH* modulation [65,85]; TLR/IL-1R-IRAK-NF-κB signaling causing altered innate immune response and inflammatory gene expression [86]	Down-regulated in plasma [49], CSF [45,48,49], and serum [30,89]
miR-195	Down-regulated in gray matter [28], hippocampus [90], iPSC-derived astrocytes from ApoE4^+/+^ AD subjects compared to ApoE3^+/+^ normal aging iPSC-derived astrocytes [90]	BACE1 [91]; APP and BACE1 [92]	ApoE-synj1-PIP2 pathway [90]	Down-regulated in CSF [25,48,90]; up-regulated in plasma [80]
miR-218	Down-regulated in gray matter [28], and temporal cortex [30]; up-regulated in dorsolateral prefrontal cortex (Brodmann area 9) and temporal cortex (Brodmann area 21/22) [52]	PTPα [93]; C3 [94]	ER-regulated tau phosphorylation [93]	Up-regulated in blood [95]

These miRNAs may regulate key genes and signaling pathways involved in the amyloidogenic pathway, Aβ clearance, tau hyperphosphorylation, and aggregation (Table 1). The transcription factor NF-κB is known to regulate multiple pathways through its different targets, among which are *APP* and *BACE1* [96], as well as several miRNAs (miR-7, miR-34a, miR-125b, miR-128, and miR-146a) listed in Table 1. The amyloidogenic pathway can be affected through down-regulation of *BACE1* by miR-9, miR-29a, miR-29b, miR-124, miR-135a, and miR-195, or Aβ clearance impairment by miR-7, miR-9, miR-16, miR-34a and miR-101. Dysregulation of miR-16, miR-124, miR-125b, miR-132, and miR-218 affects tau protein levels and/or phosphorylation, and four of them (miR-16, miR-124, miR-125b, and miR-132) are known to deregulate either amyloid β or tau pathways by acting on different targets.

The following AD-specific miRNAs were reported as potential diagnostic biomarkers in circulating fluids: miR-16 [48], miR-29a [48,49,50], miR-29b [49], miR-32 [25], miR-34a [49], miR-34c [43], miR-101 [43], miR-125b [48,49,68], miR-128 [71], miR-135a [25,84], and miR-195 [25,48]. Among these, miR-16 [43] and miR-195 [90] were proposed as biomarkers of disease progression.

Dysregulation of microRNAs may profoundly influence AD-related pathways. To interpret the functions of dysregulated miRNAs in AD, we investigated the over-represented gene ontologies (GO), annotated in miRTarBase and enriched with the 17 AD dysregulated miRNAs using the miRNA Enrichment Analysis and Annotation Tool (miEAA) (Table S1) [97]. In addition to the typical mechanisms related to AD neuropathology, GOs related to glucose dysregulation, inflammation, and immune response were also enriched [98,99]. Indeed, the list of over-represented GO with the highest numbers of occurrences included: the apoptotic process (GO0006915, q-value 0.0025003), insulin receptor signaling pathway (GO0008286, q-value 6.21 × 10^−6^), immune response (GO0006955, q-value 0.0023718), cellular response to oxidative stress (GO0034599, q-value 1.48 × 10^−4^), negative regulation of intrinsic apoptotic signaling pathway (GO2001243, q-value 4.79 × 10^−8^), positive regulation of intrinsic apoptotic signaling pathway (GO2001244, q-value 4.86 × 10^−7^), positive regulation of autophagy (GO0010508, q-value 2.55 × 10^−5^), response to cytokine (GO0034097, q-value 2.71 × 10^−5^), glucose homeostasis (GO0042593, q-value 7.17 × 10^−4^) and inflammatory response (GO0006954, q-value 0.0151675).

## 3. PD

PD is a severely debilitating neurodegenerative disease associated with motor symptoms such as slowness of movement, stiffness, tremor, and postural instability [100,101]. It is characterized by the accumulation of α-synuclein in neuronal perikarya (Lewy bodies) and neuronal processes (Lewy neurites), and the selective loss of DNs in *substantia nigra*, which results in striatal dopaminergic deficiency [101]. Current treatments aimed at preserving DNs or compensating dopamine deficit (such as levodopa and deep brain stimulation) can relieve motor symptoms but are not effective in halting or slowing disease progression [100,101].

Although the molecular mechanisms underlying PD are not fully elucidated, the progressive deterioration of vulnerable DNs arises from several cellular disturbances, including protein misfolding and aggregation, synaptic damages, apoptosis, mitochondrial dysfunctions, oxidative stress, impairment of the UPS, and neuroinflammation [102].

Multiple genetic and environmental causes of PD have been described and clarified in the last decades. Approximately 5–10% of all patients suffer from a monogenic form of PD caused by mutations in autosomal-dominant (AD)—*SNCA*, *LRRK2,* and *VPS35*—or autosomal recessive (AR)—*PINK1*, *DJ-1,* and *PARK2*—genes [103,104]. The majority of PD cases are sporadic and result from a combination of common genetic risk loci in concert with environmental factors (lifestyle, exposure to toxins, physical activity) [101].

Dysregulation of miRNA expression profiles has been described in several brain areas and fluids of PD patients, as well as in iPSCs-derived DNs generated from affected patients. Table 2 shows a list of 15 miRNAs (let-7b, miR-34b, miR-124, miR-126, miR-132, miR-133b, miR-144, miR-148b, miR-184, miR-199a, miR-204, miR-218, miR-221, miR-338, miR-425) that were found dysregulated by at least two independent studies in nervous tissues (midbrain, prefrontal cortex, amygdala, laser-micro dissected DNs, or anterior cingulate gyrus) [105,106,107,108,109,110,111,112,113,114,115,116,117,118], iPSC-derived DNs [119] and circulating fluids (CSF, plasma, serum, peripheral blood) [119,120,121,122,123,124,125,126,127,128,129,130,131,132,133,134,135,136,137,138,139,140,141,142,143,144,145,146,147,148] of PD patients, thus supporting their potential utility as biomarkers and/or therapeutic targets.

**Table 2:** Dysregulated miRNAs in human PD *post-mortem* tissues and circulating fluids.

miRNAs	PD *post-mortem* CNS/PD iPSC-Derived Neurons	Validated Target	Signaling Pathway	Circulating Fluids
let-7b	Up-regulated in DA neurons [113], and PD-specific iPSC-derived midbrain neurons [115]; down-regulated in amygdala [114]	HMGA2 [149]		Discriminating multiple system atrophy (an atypical parkinsonian disorder) from control [144]
miR-34b	Down-regulated in putamen [150], FC, amygdala, SN, and cerebellum [151]	*ADORA2A* [150]; Dj1 and Parkin [151]; α-synuclein [152]		Up-regulated in serum of multiple system atrophy patients vs PD for differential diagnosis [146]; detected in CSF [140]
miR-124	Down-regulated in prefrontal cortex of the left cerebral hemisphere [107]; up-regulated in amygdala [114]	KPNB1, KPNA3, KPNA4 [107]; p62/p38 [153]; Bim [154]; C1ql3 [155]; *ANXA5* [156]; EDN2 [157]; MEKK3 [158]; STAT3 [159]; *NEAT1*/*PDE4B* [160]; *NEAT1* [161]	Apoptosis and Autophagy [154]; AMPK/mTOR pathway [162]; MALAT1/miR-124-3p /DAPK1 signaling cascade mediating apoptosis [163]; Calpain/cdk5 pathway [164]; Hedgehog Signaling Pathway/EDN2 [157]; STL1/NF-κB axis [165]; miR-124/KLF4 axis [166]; miR-124-3p/PTEN/AKT/mTOR pathway [167]	Reduced plasma levels in PD [136]; down-regulated in plasma [137]; up-regulated in plasma [138]
miR-126	Up-regulated in DA neurons [112,113], and amygdala [114]	SP1 [168]; PLK2 [169]; LncRNA HOTAIR/RAB3IP [170]; IRS-1/PIK3R2 [171]	Insulin/IGF-1/PI3K signaling pathway [112]; GF/PI3K/AKT and ERK signaling cascades [171]	Down-regulated in CSF exosome [143], and blood [121,122]
miR-132	Down-regulated in prefrontal cortex (Brodmann Area 9) [116], and in meta-analysis from different PD brain specimens [172]; up-regulated in midbrain [117]	ncRNA MIAT [173]; ULK1 [174]; *Nurr1* [175]; GLRX [117]	SIRT1/P53 pathway [176]	Up-regulated in peripheral blood [147,148], and exosomes isolated from CSF [143]; down-regulated in serum samples [125]
miR-133b	Down-regulated in midbrain [105,106,172]	*Pitx3* [105]; FAIM [177]; RhoA [178]; SNHG14 [179]; *Gdnf* [180]	Inhibition of cell apoptosis by regulating the ERK1/2 signaling pathway [181]; Xist/miR-133b-3p/Pitx3 axis [182]	Up-regulated in plasma [120]; down-regulated in plasma [131], and serum [142]
miR-144	Up-regulated in the prefrontal cortex (Brodmann Area 9) [116], and anterior cingulate gyrus [118]; down-regulated in the prefrontal cortex of the left cerebral hemisphere [107]	KPNB1, KPNA3, and KPNA4 [107]; β-amyloid precursor protein [183]	NF-κB signaling pathway [107]	Down-regulated in serum [123]; up-regulated in CSF [124]
miR-148b	Down-regulated in the prefrontal cortex (Brodmann Area 9) [116], and amygdala [114]			Down-regulated in blood [146]
miR-184	Up-regulated in DA neurons [113] and amygdala [114]			Up-regulated in exosomes; down-regulated in plasma [145]
miR-199a	Up-regulated in the amygdala [114]; down-regulated in iPSC-derived DNs from PD patients [119]			Stage-specific biomarker in serum extracellular vesicles [133]
miR-204	Up-regulated in putamen [108]; down-regulated in amygdala [114]	SLC5A3 [184]; DYRK1A [185]		Up-regulated in CFS of Progressive Supranuclear Palsy (PSP) patients [126]; differentially expressed in plasma samples [127]; detected in CSF of patients with parkinsonian syndromes [144]
miR-218	Up-regulated in the amygdala [114], and midbrain [110]; down-regulated in the prefrontal cortex of the left cerebral hemisphere [107]	*RAB6C* [110,186]; LASP1 [187]; KPNB1, KPNA3, KPNA4 [107]; PRKN [188]	NF-κB signaling pathway [107]	Down-regulated after 1 h of deep brain stimulation [134,135]; up-regulated in plasma [145]
miR-221	Up-regulated in putamen [108], anterior cingulate gyrus [118], and amygdala [114]	LncRNA MIAT [189]; LncRNA HOTAIR [190]; LncRNA SNHG1 [191]; *DJ1* [192]; *TFR2* [193]; *FMR1* [194]	TGF-β1/Nrf2 axis [189]; miR-221/222/p27/mTOR pathway [191]	Up-regulated in plasma [120]; down-regulated in serum [128,129,130]
miR-338	Down-regulated in prefrontal cortex (Brodmann Area 9) [116], and amygdala [114]	SP1 [195]		Decreased levels in plasma extracellular vesicles [139]
miR-425	Up-regulated in putamen [108]; down-regulated in SN [109]	*RIPK1* [109]	miR-425-5p/TRAF5/NF-κB axis [196]	Able to discriminate PD from PSP [132]

Three dysregulated miRNAs (miR-34b, miR-218, miR-221) interact with PD-related genes (*DJ1*, *PRKN*, *SNCA*) and modulate their functions in different PD cellular and animal models, while others (miR-133b, miR-126, miR-132, miR-144, miR-425 and miR-124) participate in neuronal apoptosis and survival signaling pathways, as well as in autophagy mechanisms (Table 2).

The following PD-specific miRNAs have been reported as potential diagnostic biomarkers in circulating fluids: miR-126 [122], miR-144 [124], miR-184 [145], miR-204 [127] and miR-221 [120,128,129,130]. Among them, miR-144 has been proposed as an early biomarker [123]. Let-7b [144] and miR-148b [146] were proposed as biomarkers for differential diagnosis of PD from multiple system atrophy, while miR-204 [126] and miR-425 [132] from PSP. Lastly, miR-199a was proposed for the stage-specific diagnosis of PD [133].

To interpret the functions of dysregulated miRNAs in PD, we investigated the over-represented GO, annotated in miRTarBase and enriched with the 15 PD dysregulated miRNAs using miEAA (Table S2) [97]. Many of the categories are implicated in PD pathogenesis and include neuroinflammatory/immune responses (positive regulation of prostaglandin biosynthetic process GO0031394, q-value 1.36 × 10^−6^; regulation of neuroinflammatory response GO0150077, q-value 3.22 × 10^−4^; macrophage cytokine production GO0010934, q-value 3.22× 10^−4^), cell death and apoptosis (negative regulation of hydrogen peroxide-mediated programmed cell death GO1901299, q-value 2.54 × 10^−5^; positive regulation of intrinsic apoptotic signaling pathway GO2001244, q-value 4.94 × 10^−4^), and neurodevelopment (tube formation GO0035148, q-value 1.54 × 10^−4^; nerve development GO0021675 q-value 2.34 × 10^−4^; branching morphogenesis of an epithelial tube GO0048754, q-value 3.22 × 10^−4^).

## 4. ALS

ALS is a progressive neurodegenerative disease characterized by selective degeneration of upper and lower MNs, resulting in muscle weakness and atrophy, with respiratory failure and ultimately death 3–5 years after the first clinical manifestation [197]. Only a fraction of ALS cases (approximately 10%) is familiar (fALS), because of mutations in genes involved in a wide range of cellular functions, whereas the vast majority of ALS cases are sporadic (sALS) [197]. Rilutek (riluzole) and Radicava (edaravone) are the only two drugs approved for ALS, which only slightly slow disease progression [198].

Understanding the etiopathogenesis of ALS is crucial for the implementation of effective therapies that are urgently needed. ALS is considered to have a complex etiology involving multiple genes and environmental factors. Among the implicated pathological processes are protein aggregation, glutamate excitotoxicity, defects in stress response, mitochondrial dysfunction, protein aggregation, altered axonal transport, and aberrant RNA metabolism [199,200,201]. The role of this last, in particular, seems particularly central when considering that several ALS-linked genes, such as *TARDBP* or *FUS*, are key components of coding and noncoding RNA processing machinery [17,202,203,204,205,206,207,208].

The role of miRNAs in ALS pathology is highlighted by several studies describing dysregulated miRNAs in the spinal cord, brain, blood, CSF, and iPSCs of ALS patients [209,210,211,212,213,214,215]. Here we focused our attention on a list of 9 miRNAs (miR-9, miR-124, miR-142, miR-146a, miR-155, miR-218, miR-133a, miR-133b, miR-338), which were found differentially expressed in both tissues (cortex and spinal cord) and fluids of ALS patients. Four of these (miR-9, miR-218, miR-133a, and miR-133b) were also implicated in iPSC-derived MNs of ALS patients, further supporting their potential utility as biomarkers and/or therapeutical targets (Table 3).

**Table 3:** Dysregulated miRNAs in human ALS *post-mortem* tissues or patient-specific iPSC-derived MNs and circulating fluids.

miRNAs	ALS *post-mortem* CNS/ALS iPSC-Derived Neurons	Validated Target	Signaling Pathway	Circulating Fluids
miR-9	Down-regulated in lumbar motor neurons [202,215,216]; dysregulated in ALS-specific iPSC-derived MN lines [217,218]	*NEFL* [215,216]; *PRPH* [218]; FoxP1 [219]; PAK4 [220]	Neuronal transcription programs, neurofilaments aggregate formation [215,216,221]	Increased in peripheral leukocytes from ALS patients [222]
miR-124	Down-regulated in spinal cord [202,214]	Sox2, Sox9 [223]	Immune responses, neuroinflammation, neuronal development, synaptic plasticity, neurodegeneration [224,225,226]	Dysregulated in the CSF and leukocytes of ALS patients [222,227,228]
miR-133a/b	Down-regulated in spinal cord tissue [212,229], and ALS-specific iPSC-derived MN [210]	*FAS*, *CD4*, *EIF2C4/AGO4*, *CCL2*, and *AQP1* [212]	Cell death, defense response, immune response, and inflammation [212]	Up-regulated in serum [230,231]
miR-142	Up-regulated in spinal cord tissue [212,229]	CAMK2A [232]; Vimentin [233]; IL-6 [234]; CDKN1B, TIMP3 [235]; NRF2 [227,236,237]	Cell death, defense responses, immune responses and inflammation [212,238]	Dysregulated in CSF of ALS patients [227,238,239,240]
miR-146a	Dysregulated in spinal cord tissue [215,216,229]	*NEFL* [215,216]	Neurofilaments aggregate formation [215,216]; neuroinflammation [241]	Up-regulated in blood plasma from ALS/MND patients [242]
miR-155	Up-regulated in spinal cord [212,214,229]	SHIP1 [229];SOCS1 [243];SMAD2 [244]; SMAD5 [245]; TGF-β [246]	Cell death, defense responses, immune responses, and inflammation [212]	Increased in peripheral monocytes from ALS patients [247]
miR-218	Down-regulated in spinal cord tissue [212,229]; up-regulated in ALS-specific iPSC-derived MN [248]	Kcnh1 [249]; SLC1A1, SLC1A2 [248]; *Tead1*, *SLC6A1*, *BCL11A*, *Lhx1* and *FoxP2* [250]	Development, membrane excitability, NMJ synaptic connections [249]	Down-regulated in peripheral blood, CSF, serum and neuromuscular junction of ALS patients [251]
miR-338	Up-regulated in spinal cord tissue [252], and motor cortex samples [209,212]	*ATP5G1* [253]	Apoptosis, oligodendrocyte differentiation, maturation, mitochondrial function [254]	Up-regulated in peripheral blood, CSF, serum and neuromuscular junction of ALS patients [222,251,252,254,255]

Most ALS-related miRNAs mentioned above regulate the expression of genes involved in oxidative stress and neuroinflammation, whereas two of them (miR-155 and miR-142) are predicted regulators of ALS-related gene transcripts (*TARDBP*, *UBQLN2*, *KIF5A*, and *C9orf72*). In particular, miR-155 promotes tissue inflammation and macrophage inflammatory responses by targeting several immune response-related gene transcripts, including *SOCS1*, *C/EBPβ*, *TGF-β*, *SMAD2*, and *SMAD5* [243,244,245,256,257]. Increased levels of miR-155 were found both in spinal cord tissue and peripheral monocytes of ALS patients and its inhibition increases survival time and disease duration in a murine ALS model, supporting the possibility to use this miRNA as a therapeutical target [212,214,229,247] (Table 3).

MiR-142 is an important regulator of neuronal viability and apoptosis. Its inhibition produces neuroprotective effects by reducing neuronal injury and oxidative stress via the IL-6 and Nrf2/ARE signaling pathways and modulates axonal transport and mitochondrial activity in MNs by targeting vimentin and other intermediate filament types [232,233,234,235,258,259].

Functional enrichment analysis of the 9 dysregulated miRNAs in *post-mortem* tissues and circulating fluids of ALS patients produces a list of over-represented GO terms, many of which were previously implicated in ALS pathogenesis (Table S3) [97]. Among these are multiple processes involved in neuroinflammatory/immune responses, such as epidermal growth factor receptor signaling activity (GO0005006, q-value 1.07 × 10^−6^), regulation of neuroinflammatory response (GO0150077, q-value 2.74 × 10^−6^), activation of phospholipase A2 activity by calcium-mediated signaling (GO0043006, q-value 3.42 × 10^−6^), positive regulation of interleukin-17 biosynthetic process (GO0045380, q-value 3.42 × 10^−6^), regulation of astrocyte activation (GO0061888, q-value 3.42 × 10^−6^), NAD-dependent histone deacetylase activity (GO0017136, q-value 1.34 × 10^−5^), negative regulation of ERBB signaling pathway (GO1901185, q-value 1.13 × 10^−5^), positive regulation of cytokine activity (GO0060301, q-value 1.44 × 10^−5^), C-X-C motif chemokine 12 receptor activity (GO0038147, q-value 1.73 × 10^−5^), CXCL12-activated CXCR4 signaling pathway (GO0038160, q-value 1.73 × 10^−5^), positive regulation of protein kinase C activity (GO1900020, q-value 2.06 × 10^−5^), neutrophil apoptotic process (GO0001781, q-value 2.06 × 10^−5^), and positive regulation of apoptotic DNA fragmentation (GO1902512, q-value 2.27 × 10^−5^).

## 5. Common Dysregulated miRNAs in AD, PD, and ALS

In the previous sections, we reported the altered expression of specific miRNA molecules in nervous tissue and fluids of patients with AD, PD, and ALS. Although each of these NDs has its own unique clinical aspects, they share common pathological features and etiopathogenetic mechanisms such as inflammation or apoptosis. Identification of commonly dysregulated miRNAs may provide useful insights into the implicated molecular pathways thus unrevealing novel potential drug targets.

**Figure 1:**
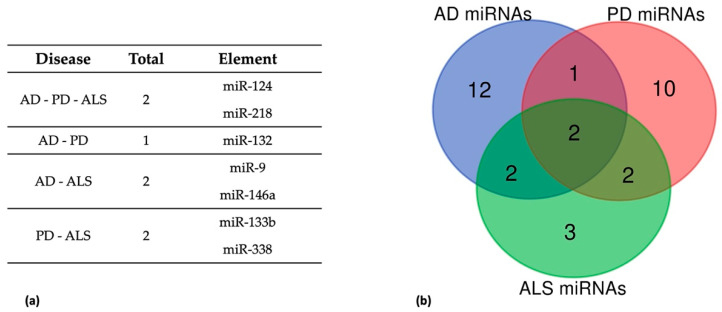
Dysregulated miRNAs in AD, PD, and ALS. List of commonly dysregulated miRNAs (**a**) and Venn diagram (**b**) of dysregulated miRNAs in the three NDs (Table 1, Table 2 and Table 3).

**Figure 2:**
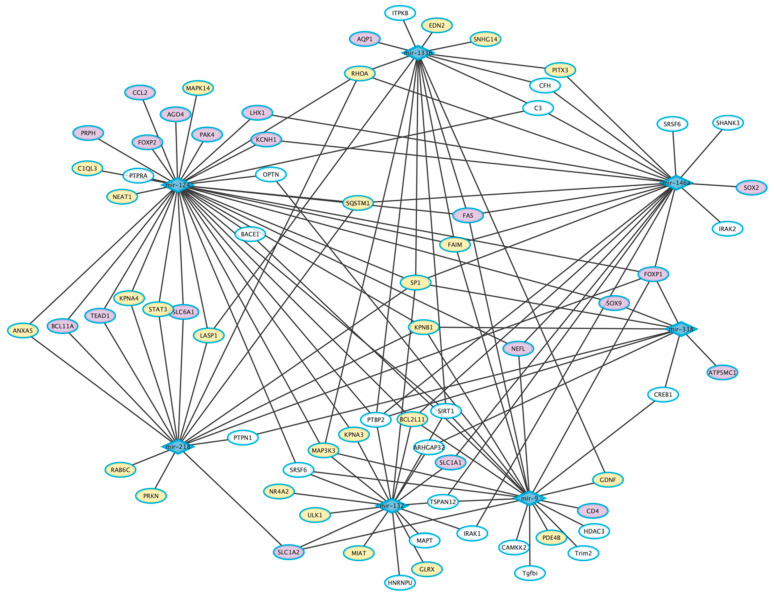
Interaction network of dysregulated miRNAs and their targets. The network was constructed using miRNet [260] and the miRNAs identified in this review as dysregulated in AD, PD, and ALS as an input list together with their disease-associated targets shown in Table 1, Table 2 and Table 3. Network visualization was obtained using the Cytoscape tool [261]. The most interconnected node (hub) is represented by miR-124 with a degree of connection of 36, while a degree of connection of 16 has been calculated for miR-218 which is also common to the three NDs pathologies. The blue diamond icons represent the dysregulated miRNAs, while ellipses represent target genes and are colored based on their disease association (yellow = PD; purple = ALS; light blue = AD).

Using the lists of commonly dysregulated miRNAs in human *post-mortem* nervous tissues and circulating fluids of AD, PD, and ALS patients (Table 1, Table 2 and Table 3), we identified 7 commonly dysregulated miRNAs (miR-9, miR-124, miR-218, miR-132, miR-133b, miR-338, miR-146a) (Figure 1). In particular, altered expression of miR-124 and miR-218 was reported in all the three NDs (Figure 1a). MiR-133b and miR-338 were dysregulated in PD and ALS, miR-132 in both PD and AD, while miR-9 and miR-146a in AD and ALS (Figure 1a). The regulatory interaction network among these overlapping miRNAs and their corresponding disease-associated targets shows a high level of interconnectedness, with miR-124 as the most interconnected node (hub) in the network and commonly dysregulated miRNA for the three NDs pathologies (Figure 2). This suggests the possibility to target a single miRNA and affect multiple pathogenic pathways.

In the next sections, we will describe these commonly dysregulated miRNAs and review their potential role and main targets.

### 5.1. Dysregulated miRNAs in AD, PD, and ALS

Several studies reported dysregulation of miR-124 in AD, PD, and ALS [225,262] (Table 1, Table 2 and Table 3). This represents one of the most abundant miRNAs in CNS and plays an important role in neuronal survival, autophagy, mitochondrial dysfunction, synapse morphology, oxidative damage, and neuroinflammation by modulating the activity of downstream factors [263] (Table 1, Table 2 and Table 3, Figure 1). Specifically, in AD miR-124 modulates both Aβ production by targeting *BACE1* [58,60] *APP* [59] and tau phosphorylation levels through PTPN1 signaling [62], and its decrease was detected in the CSF of patients with AD, supporting its role as a potential diagnostic biomarker in AD [43] (Table 1, Figure 2). Reduced plasma miR-124 levels support its potential utility as a diagnostic biomarker in the early stage of PD [136] (Table 2). In particular, aberrant expression of miR-124 in DNs leads to mitochondrial damage and cell death by targeting many key components of AMPK/mTOR, NF-κB, and p25/CDK5 pathways, including p62/p38, STAT3, KPNB1, and Calpains 1–2 [107,136,153,158,159,162,164,264,265,266] (Table 2, Figure 2). In addition, miR-124 interacts with the modulator of BCL2-interacting mediator of cell death (Bim), whose suppression leads to reduction of Bax translocation to mitochondria and lysosomes, attenuating apoptosis and autophagosome accumulation [154] (Table 2, Figure 2). In ALS, miR-124 exerts a neuroprotective role in transgenic mice, by targeting *Sox2* and *Sox9*, which encode two important regulators of neuronal and glial differentiation (Table 3, Figure 2) [223,225]. Differential expression of this miRNA can also be detected in both the spinal cord and leukocytes of sALS patients (Table 3) [222,227,228].

In addition to PD, AD, and ALS (Table 1, Table 2 and Table 3, Figure 1), miR-218, has been associated with neuropsychiatric disorders and other NDs [135,249,267,268]. In AD it is considered a potential peripheral biomarker [95] and was shown to regulate learning and memory in a mice AD model [94] and to affect the homeostasis between phosphorylated and dephosphorylated tau proteins [93] (Table 1, Figure 2). In PD models, miR-218 plays a role in modulating the NF-κB inflammatory signaling pathway, by influencing the activity of three importins, KPNB1, KPNA3, and KPNA4 [107], and interacts with the PD related gene *PRKN* [269], leading to mitochondrial dysfunction through the autophagic pathway [188] (Table 2, Figure 2). In addition, altered levels of miR-218 were found in brain regions and blood of PD patients [145] and were also associated with therapeutic brain stimulation [134,135] (Table 2). Dysregulation of miR-218 was also observed in ALS patients and animal models [212,229,248,251] (Table 3). A direct target of miR-218 in MNs is the voltage-gated potassium channel Kv10.1, whose upregulation was associated with an abnormal neuronal activity and excitability of MNs [249] (Table 3, Figure 2). It also targets EAAT2 (encoded by *SLC1A2)*, an astrocytic glutamate excitatory amino acid transporter, that carries glutamate back into the cell after neurotransmission [248] and, when mutated, leads to impairment of glutamate levels, promoting post-synaptic neuronal cell death [270] (Table 3, Figure 2).

### 5.2. Dysregulated miRNAs in AD and PD

MiR-132 has been linked to several neurophysiological processes such as neuronal differentiation, migration and maturation, synaptic transmission, plasticity, and neuroprotection [271,272]. In particular, it represents one of the most-studied miRNAs in AD and, together with its downstream molecular targets (HDAC3, ITPKB, p250GAP, HNRNPU, PTBP2, and SIRT1), is involved in the regulation of two AD pathological hallmarks: tau and Aβ [72,73,74,75,76,77,78] (Table 1, Figure 2). Dysregulated expression levels of this miRNA were found in the brain and CSF of AD patients and correlated with disease progression, supporting its use as an early biomarker (Table 1) [43]. MiR-132 was also proposed as a good candidate for monitoring PD progression as well as response to various therapeutic approaches [125,143,152] (Table 2). Upregulation of this miRNA was associated with neuroinflammation, microglial activation, and DNs neurodegeneration [117,148] (Table 2, Figure 2).

### 5.3. Dysregulated miRNAs in AD and ALS

Among miRNAs differentially expressed in brain tissues and fluids of AD and ALS patients, miR-9 is a brain-specific miRNA that has demonstrated great potential as a biomarker (Table 1 and Table 3, Figure 1). Its levels were reduced in the blood of LOAD patients [42] and correlated with disease severity [43] as was ell response to treatment in primary neurons (Table 1). In particular, the synapse-enriched miR-9 [40] regulates different AD-related genes (*BACE1*, *CREB*, *OPTN*, and *CAMKK2*) influencing Aβ production and autophagy [34,38,39,40], together with other targets related to neurotrophic proteins [41,273] (Table 1, Figure 2). MiR-9 plays an important role in regulating MNs development and its differential expression in ALS leukocytes supports its role as a diagnostic biomarker [218,219,220] (Table 3). Since it is known to interact with the 3′-UTRs of *NEFL* and *PRPH* and Pak4, its dysregulation may affect cell-cell junctions and axonal transport, leading to MN degeneration [17,218,220,274] (Table 3, Figure 2). Similar pathogenic mechanisms may follow the dysregulation of the NF-κB-sensitive miR-146a, implicated in the formation of pathological neurofilamentous aggregates [215,216,229], neuroinflammation, and immune response [55,65,85,86,87,88] (Table 1 and Table 3, Figure 1). Differential expression of this miRNA in plasma and CSF of AD and ALS patients [65,85] supports its role as a potential biomarker [242] (Table 1 and Table 3).

### 5.4. Dysregulated miRNAs in PD and ALS

As anticipated, miR-133b and miR-338 are dysregulated in PD and ALS (Table 2 and Table 3, Figure 1). In particular, circulating miR-133b levels are altered in the early stages of PD [120] (Table 2). MiR-133 influences the maturation, function, and apoptosis of DNs [105,275,276,277,278] and also regulates RhoA, a protein modulating α-Synuclein expression [178,279] (Table 2, Figure 2). Increased serum level of miR-133b in ALS may influence skeletal muscle development [203,280] and neuromuscular junction maintenance/reinnervation [230,231] and targets several ALS-related genes, such as *CCL2*, *CD4*, *FAS*, *EIF2C4*/*AGOA* and *AQP1* [212] (Table 3, Figure 2).

In PD, miR-338 has been functionally linked to DNs survival and its decrease in plasma extracellular vesicles has been proposed as a potential diagnostic biomarker [139] (Table 2). In ALS, this miRNA was found differentially expressed in blood, CFS, serum, and spinal cord, and its use as an effective early biomarker has been considered [222,251,252,254,255] (Table 3). From a functional point of view, miR-338 modulates the expression of COXIV and ATP synthase [281], as well as the ALS-related genes *ARHGEF28* (involved in the aggregation of low molecular weight neurofilaments) and *VAPB* (involved in protein misfolding and ER-associated aggregates) [282,283]. Moreover, ectopic expression of miR-338 mediated by FoxO3a may play a critical role in reducing cell survival by directly suppressing the expression of *NRP1* [284] (Table 3, Figure 2).

## 6. ASOs-Based miRNA Therapies

The leading approach against inappropriate miRNA expression is based on ASOs. ASOs-therapies are used to directly modulate the expression of mRNAs or miRNAs. They are based on single-stranded oligonucleotides forming a complementary heteroduplex with the targeted mRNA, complementary double-stranded oligonucleotides miming endogenous miRNAs, or single-stranded that inhibit miRNAs [285]. These molecules can be used to mimic (agomir) or, more often, inhibit (antagomir) specific miRNAs [285], and simultaneously affect the expression of multiple proteins [13,286]. To allow adequate bio-distribution of therapeutic ASOs to the brain and circumvent the BBB, they can be directly delivered to the CSF (ICV or intrathecal) [20,285]. Taking advantage of their ability to regulate the expression of multiple genes, therapies involving miRNAs offer this peculiar opportunity to be used in different pathologies.

Although no miRNA-based ASOs have yet entered the clinical phase in AD, PD, or ALS, some miRNA-based therapies have been pre-clinically tested in vitro or in vivo, and showed promising results either in AD [13,285], PD [269,277], or ALS [205,287,288]. One of the most interesting examples is miR-124, which is dysregulated in all three pathologies (Figure 1). In AD, miR-124 mimic was used to regulate BACE1 and alleviate cell death induced by Aβ neurotoxicity [289], and reduce *APP* gene expression [59], while the use of a miR-124 antagomir resulted in the attenuation of tau phosphorylation and increased PTPN1 levels [62]. In MPTP-induced mouse models of PD, the use of a miR-124 mimic promotes neuronal proliferation and suppression of neuronal apoptosis via the Hedgehog signaling pathway [157]. The over-expression of miR-124 significantly reverses the loss of DNs and striatal DA, and reduces autophagosome accumulation and lysosomal depletion in MPP(+)-intoxicated SH-SY5Y cells [154]. Exogenous delivery of miR-124 attenuates microglia activation in SN and apoptotic cell death in midbrain DA of MPTP-treated mice in vivo [153,158]. In addition, polymeric nanoparticles (NPs) have been used to deliver miR-124 to specific regions of the brain [290,291]. Normalization of miR-124 level in ALS cellular models by using miR-124-targeting drugs attenuates inflammatory responses by inhibiting the NF-kB signaling pathway and preventing neuronal death [225,226].

Neuroprotective effects were obtained with antagomir inhibition of miR-218, a miRNA dysregulated in AD, PD, and ALS patients. In vivo ASO-mediated inhibition of miR-218 has anti-inflammatory, anti-apoptotic, and antioxidant effects in ALS model mice by attenuating the loss of a key glutamate transporter, the excitatory amino acid transporter Slc1a2 [248].

Among miRNAs dysregulated in AD and PD (Table 1 and Table 3, Figure 1), miR-132 showed promising therapeutic properties in AD mouse models, where treatment with miR-132 mimics restores memory function [79] and reduces phosphorylation of tau and Aβ [72,73]. Similar therapeutic effects were also obtained by inhibiting miR-9 and miR-146a, two miRNAs that are frequently dysregulated in AD and ALS (Table 1 and Table 2, Figure 1). Indeed, miR-9 antagomir rescues upregulation of BACE1 [38], and promotes cognition and autophagic clearance of Aβ [39] in AD mice. ASO-based miR-146a mimic improves behavioral and cognitive dysfunction while attenuating neuroinflammation, glial activation, Aβ deposition, and tau phosphorylation in mice hippocampus [88].

## 7. Conclusions

The recognition that inappropriate production of individual miRNAs may contribute to NDs has invigorated interest in these molecules and hope for new diagnostic methods and therapeutical approaches. While the pathogenic role of inappropriate miRNA expression is being characterized, different strategies to mimic or inhibit these miRNAs by ASOs have been effectively tested in pre-clinical models of NDs. Although delivery of these ASOs therapies to brain cells remains a key obstacle, the successful translation from in vitro and experimental animal studies into clinical practice may soon allow the development of effective drugs.

## Data Availability

The data presented in this study are available in the supplemental material.

## Supplementary Materials

The following supporting information can be downloaded at: https://www.mdpi.com/article/10.3390/jpm12050770/s1, Table S1. GOs enrichment for dysregulated miRNAs in AD human nervous tissues and circulating fluids. Table S2. GOs enrichment for dysregulated miRNAs in PD human nervous tissues and circulating fluids. Table S3. GOs enrichment for dysregulated miRNAs in ALS human nervous tissues and circulating fluids.

## Abbreviations

AD = Alzheimer’s disease; Add1 = Adducin 1; ADORA2A = Adenosine A2a Receptor; AGO4 = Argonaute RISC Component 4; ALS = Amyotrophic Lateral Sclerosis; AMPK = AMP-activated protein kinase; ANXA5 = Annexin A5; APOE = Apolipoprotein E; APP = Amyloid Beta Precursor Protein; AQP1 = Aquaporin 1; ARHGEF28 = Rho guanine nucleotide exchange factor 28; ASOs = antisense oligonucleotides; ATP5G1 = ATP Synthase Membrane Subunit C Locus 1; Aβ = amyloid-beta; miRNA = microRNA; BACE1 = Beta-Secretase 1; Bax = BCL2 Associated X, Apoptosis Regulator; BBB = blood-brain barrier; Bim = Bcl-2-like 11; C1ql3 = complement C1q like 3; C3 = Complement C3; CAMK2A = Calcium/Calmodulin Dependent Protein Kinase II Alpha; CCL2 = C-C Motif Chemokine Ligand 2; CDK5 = Cyclin dependent kinase 5; CDKN1B = Cyclin Dependent Kinase Inhibitor 1B; CDKN2A = Cyclin Dependent Kinase Inhibitor 2A; CEBPB = CCAAT Enhancer Binding Protein Beta; CEBPD = CCAAT Enhancer Binding Protein Delta; CFH = Complement Factor H; CHMP2B = Charged Multivesicular Body Protein 2B; ciRS-7= circular RNA 7; CNS= central nervous system; CNV = copy number variation; COXIV = Cytochrome C Oxidase Subunit 4; CREB = cAMP Responsive Element Binding Protein 1; CSF = cerebrospinal fluid; DA = dopamine; DAPK1 = Death Associated Protein Kinase 1; KLF4 = Kruppel Like Factor 4; DJ-1 = PARK7 Parkinsonism associated deglycase; DN = Dopaminergic neurons; DUSP6 = Dual Specificity Phosphatase 6; EAAT2 = Excitatory amino acid transporter 2; EDN2 = Endothelin 2; EOAD = early-onset AD; ER = endoplasmic reticulum; ERK = Extracellular Signal-Regulated Kinase; fAD = familial AD; FAIM = Fas Apoptotic Inhibitory Molecule; fALS = familial ALS; FAS = Fas Cell Surface Death Receptor; FMR1 = FMRP translational regulator 1; Foxo3a = Forkhead box O3; FoxP1 = Forkhead Box P1; FUS = Fused in Sarcoma; Gdnf = Glial cell derived neurotrophic factor; GF = growth factor; GO = Gene Ontology; HDAC3 = Histone Deacetylase 3; HMGA2 = high mobility group A2; HNRNPK = Heterogeneous Nuclear Ribonucleoprotein K; HOTAIR = HOX antisense intergenic RNA; IGF-1 = insulin-like growth factor 1; IL-1R = Interleukin 1 receptor; IL-1β = Interleukin 1 Beta; IL-6 = Interleukin 6; iPSC = induced pluripotent stem cells; IRAK-1 = Interleukin 1 Receptor Associated Kinase 1; IRAK-2 = Interleukin 1 Receptor Associated Kinase 2; IRS-1 = Insulin receptor substrate 1; ITPKB = Inositol-Trisphosphate 3-Kinase B; Kcnh1 = Potassium Voltage-Gated Channel Subfamily H Member 1; KIF5A = Kinesin Family Member 5A; KPNA3 = Karyopherin Subunit Alpha 3; KPNA4 = Karyopherin Subunit Alpha 4; KPNB1 = Karyopherin Subunit Beta 1; LASP1 = LIM And SH3 Protein 1; Lhx1 = LIM Homeobox 1; LncRNA = Long non-coding RNA; LOAD = late-onset AD; LRRK2 = Leucine Rich Repeat Kinase 2; MALAT1 = Metastasis Associated Lung Adenocarcinoma Transcript 1; MAPT = Microtubule Associated Protein Tau; Mcpip1 = Monocyte Chemotactic Protein-Induced Protein 1; MECP2 = Methyl-CpG Binding Protein 2; MEKK3 = Mitogen-activated protein kinase kinase kinase 3; MIAT = myocardial infarction–associated transcript; MN = motor neuron; MPTP = 1-methyl-4-phenyl-1,2,3,6-tetrahydropyridine;MPP = 1-methyl-4-phenylpyridinium; mTOR = Mechanistic Target Of Rapamycin Kinase; NDs = neurodegenerative diseases; NEAT1 = nuclear paraspeckle assembly transcript 1; PDE4B = Phosphodiesterase 4B; NEFL = Neurofilament Light Chain; NF-kB = nuclear factor kappa-light-chain-enhancer of activated B cells; NFTs = neurofibrillary tangles; NMJ = neuromuscular junction; NP = nanoparticle; NR2A= N-methyl-D-aspartate (NMDA) receptor 2A; FMRP = fragile X mental retardation protein; PPAR = peroxisome proliferator-activated receptor; Nrf2 = Nuclear factor erythroid 2-related factor 2; NRP1 = Neuropilin 1; Nurr1 = nuclear receptor related 1 protein; OPTN = Optineurin; p250GAP = p250 GTPase-activating protein; PAK4 = Serine/threonine-protein kinase; PARK2 = Parkin; PD = Parkinson’s disease; PI3K = Phosphatidylinositol 3-kinases; PIK3R2 = Phosphatidylinositol 3-kinase regulatory subunit beta; PINK1 = PTEN Induced Kinase 1; PIP2 = phosphoinositol biphosphate; Pitx3 = Paired Like Homeodomain 3; PLK2 = Polo-like Kinase 2; PPP1CA = Protein Phosphatase 1 Catalytic Subunit Alpha; Bcl = B-cell lymphoma; PRKN = Parkin RBR E3 Ubiquitin Protein Ligase; PRPH = Peripherin; PSEN1 = Presenilin 1; PSEN2 = Presenilin 2; PSP = Progressive supranuclear palsy; PTBP2 = Polypyrimidine Tract Binding Protein 2; PTEN = Phosphatase and tensin homolog; AKT = RAC-alpha serine/threonine-protein kinase; PTPN1 = Protein Tyrosine Phosphatase Non-Receptor Type 1; PTPα = protein tyrosine phosphatase alpha; RAB3IP = Ras-related protein Rab-3A (RAB3A)-interacting protein; RhoA = Ras Homolog Family Member A; RIPK1 = Receptor Interacting Serine/Threonine Kinase 1; Rock2 = Rho-associated protein kinase 2; sAD = sporadic AD; sALS = sporadic ALS; SHANK3 = SH3 And Multiple Ankyrin Repeat Domains 3; SHIP1 = SH2 domain-containing inositol phosphatase 1; SIRT1 = Sirtuin 1; SLC1A1 = Solute Carrier Family 1 Member 1; SLC1A2 = Solute Carrier Family 1 Member 2; SLC5A3 = Solute Carrier Family 5 Member 3; SLC6A1 = Solute Carrier Family 6 Member 1; SMAD2 = SMAD Family Member 2; SMAD5 = SMAD Family Member 5; SNCA = Synuclein Alpha; SNHG1 = Small Nucleolar RNA Host Gene 1; SNHG14 = Small Nucleolar RNA Host Gene 14; SNpc = substantia nigra pars compacta; SOCS1 = Suppressor Of Cytokine Signaling 1; SOD1 = Superoxide Dismutase 1; Sox2 = SRY-Box Transcription Factor 2; Sox9 = SRY-Box Transcription Factor 9; SQSTM1 = sequestosome 1; Srsf6 = Serine And Arginine Rich Splicing Factor 6; STAT3 = Signal Transducer And Activator Of Transcription 3; Synj1 = Synaptojanin 1; TDP-43 = TAR DNA Binding Protein 43; Tead1 = TEA Domain Transcription Factor 1; TFR2 = Transferrin Receptor 2; TGF = Transforming Growth Factor; THBS1 = Thrombospondin 1; TIMP3 = TIMP Metallopeptidase Inhibitor 3; TLR = Toll-Like Receptor; TRAF5 = TNF Receptor Associated Factor 5; TREM2 = Triggering Receptor Expressed On Myeloid Cells 2; TRIM2 = Tripartite Motif Containing 2; UBE2A = Ubiquitin Conjugating Enzyme E2 A; UBQLN2 = Ubiquilin 2; UCHL1 = Ubiquitin C-Terminal Hydrolase L1; ULK1 = Unc-51 like kinase 1; UPS = ubiquitin-proteasome system; UTR = untranslated region; VAPB = Vesicle-associated membrane protein-associated protein B/C; VPS35 = VPS35 Retromer Complex Component; Xist = X Inactive Specific Transcript.
